# Development of a fluorescent probe-based recombinase polymerase amplification assay for rapid detection of Orf virus

**DOI:** 10.1186/s12985-015-0440-z

**Published:** 2015-12-02

**Authors:** Yang Yang, Xiaodong Qin, Guangxiang Wang, Yuen Zhang, Youjun Shang, Zhidong Zhang

**Affiliations:** State Key Laboratory of Veterinary Etiological Biology, Lanzhou Veterinary Research Institute, Chinese Academy of Agriculture Sciences, Xujiaping 1, Lanzhou, 730046 Gansu China; The Medical School, University College London, Gower Street, London, WC1E 6BT UK

**Keywords:** Recombinase polymerase amplification assay, RPA assay, Orf virus, Small ruminants

## Abstract

**Background:**

Orf virus (ORFV) is the causative agent of Orf (also known as contagious ecthyma or contagious papular dermatitis), a severe infectious skin disease in goats, sheep and other ruminants. The rapid detection of ORFV is of great importance in disease control and highly needed. A isothermal molecular diagnostic approach, termed recombinase polymerase amplification (RPA), is considered as an novel and rapid alternative techonology to PCR assay.

**Results:**

In the present study, a novel fluorescent probe based on RPA assay (ORFV exo RPA assay) was developed. The developed ORFV exo RPA assay was capable of as low as 100 copies of ORFV DNA /reaction and was highly specific, with no cross-reaction with closely related viruses (capripox virus, foot-and-mouth disease virus or peste des petits ruminants virus). Further assessment with clinical samples showed that the developed ORFV exo RPA assay has good correlation with qPCR assays for detection of ORFV.

**Conclusions:**

These results suggest that the developed ORFV exo RPA assay is suitable for rapid detection of ORFV.

**Electronic supplementary material:**

The online version of this article (doi:10.1186/s12985-015-0440-z) contains supplementary material, which is available to authorized users.

## Background

The Orf virus (ORFV) is a prototype member of the Parapoxvirus genus within the Poxviridae family and the viral genome consists of a linear double-stranded DNA (137–139 kbp in length). ORFV is the causative agent of contagious ecthyma or contagious papular dermatitis (Orf) in goats, sheep and other ruminants, with a worldwide distribution and significant financial importance [1, 2]. The clinical symptoms of Orf manifest as the formation of papules, vesicles and growing scabs on the lips and muzzle of infected animals [3, 4]. Orf is usually more severe in goats than in sheep and the morbidity of the disease may reach up to 100 %. The mortality is usually not high but can reach up to 90 % in susceptible flocks of young sheep in an epidemic situation [5, 6]. Furthermore, the disease has zoonotic potential which adds to the significance of the disease to public health. Therefore, the rapid detection of ORFV is of great importance in disease control and highly needed.

Although clinical signs relating to the oral mucosa and lips are indicative of Orf, a laboratory diagnosis is necessary for confirmation and epidemiological investigations. Traditional laboratory diagnostic methods include virus isolation, electron microscopy and serum neutralization [7]. However, these methods are laborious and time-consuming. Detection of serum antibody is also not effective because of the cell-mediated nature of ORFV immunity. Recently, a number of nucleic acid-based methods (such as PCR, PCR-restriction enzyme digestion and real-time PCR) have been developed for accurate and rapid diagnosis of Orf [7–10]. Among these assays, real-time quantitative PCR (qPCR) has shown to be a highly sensitive and specific assay for detection and quantification of ORFV in clinical samples and is able to differentiate from related viruses. However, qPCR assay relies on specialized and expensive thermocycling machines, as a result it is difficult to be used as a “pen-side” test and in endemic areas with low resources.

In recent years, a novel and rapid isothermal molecular diagnostic approach, termed recombinase polymerase amplification(RPA), has been developed as an alternative to PCR assay. The RPA technology employs three core enzymes: a recombinase, a single-stranded DNA-binding protein (SSB) and a strand-displacing polymerase. During the RPA reaction, recombinases first pairs oligonucleotide primers with homologous sequences in duplex DNA. SSB binds to displaced strands of DNA and prevents the primers from being displaced. Then the strand-displacing polymerase begins DNA synthesis at sites where the primer has bound to the target DNA. Real-time detection can be achieved by adding exonuclease III and exo probes to the reaction mixture. Importantly, RPA reaction can be performed between 37 ~ 42 °C without requirement of any sophisticated equipment. In comparison to loop-mediated isothermal amplification (LAMP) [11, 12], which requires a larger set of six primers, a higher temperature (62 °C) and a longer run time, RPA is more rapid and simpler to run, which just needs a pair of primers [13–15], a lower temperature (37 to 42 °C) and a shorter run time (less than 20 min). Since its initial development in 2006, RPA technology has been successfully used for rapid detection of various pathogens [16–21]. In the present study, a fluorescent probe-based RPA assay has been developed and evaluated for rapid detection of ORFV. To the best of our knowledge, a RPA assay has not been developed for detection of ORFV yet. After determination of the sensitivity and specificity of the assay, clinical samples from sheep were tested and compared with results from the corresponding qPCR assay.

## Methods

### Virus and cells

All viruses used in this study were preserved in our laboratory: ORFV/Vaccine/CHA, ORFV/Gansu/CHA, ORFV/HB/CHA; Capripox virus CHA vaccine strain, Capripox virus/Henan/CHA; peste des petits ruminants virus (PPRV) Nigeria 75/1; foot-and-mouth disease virus (FMDV)/O/CHA, FMDV/A/CHA and FMDV/Asia 1 /CHA. A549 cells were preserved in our laboratory and cultured in minimal essential medium (MEM) containing 10 % fetal bovine serum (FBS) at 37 °C, 5 % CO_2_.

### Sample preparation

Twenty two field samples (*n* = 22) were collected from suspected goats of Orf and eight nasal swabs collected from eight experimentally infected sheep. Swabs were placed immediately after collection in 1 ml phosphate-buffered saline (PBS) and stored at −80 °C until used. To prepare ORFV-spiked tissues lysates, ORFV-free tissues samples of skin, lymphatic nodes liver, lungs, stomach and kidney (*n* = 24, three each tissue) were collected from four healthy sheep. ORFV is an epitheliotropic virus, therefore sample of skin tissue was selected. In addition, samples of other tissues were also chosen because various types of tissues are often received in the field diagnostics for differential diagnosis including non-epitheliotropic viruses such as PPRV, so it would be critical to access the compatibility of the developed RPA assay with different tissues matrix. 10 % (w/vol) tissue suspensions were then prepared by homogenizing tissue samples in PBS. Following a brief centrifugation, the homogenized tissue samples were spiked with 10^4^ TCID_50_ of ORFV/HB/CHA and stored at −80 °C until used. To prepare ORFV infected A549 cells (human lung adenocarcinoma cells, provided by our laboratory), the A549 cells were seeded at 1 × 10^6^ cells/well in 6 well-plates and cultured overnight in MEM containing 10 % FBS and incubated overnight at 37 °C, 5 % CO_2_. Growth medium was removed and 100 μL of ORFV/Vaccine/CHA, ORFV/Gansu/CHA and ORFV/ HB/CHA in medium with 2 % FBS was added into their own wells, respectively. Cells in negative control well were maintained in medium with no ORFV. After 1 h of adsorption at 37 °C, the cells were washed gently three times with serum free medium and then maintained in MEM containing 2 % FBS at 37 °C, 5 % CO_2_. The cells were harvested at 12, 24, 36, 48 and 60 h after inoculation (hpi) and stored at −80 °C until use.

### DNA extraction

Total DNA was extracted from samples using high pure viral nucleic acid kit (Roche) according to the manufacturer’s instructions and eluted in a final volume of 50 μL. Extracted DNA was stored at −80°Cuntil further use.

### Generation of DNA standard

The ORFV DNA polymerase gene segments (305 bp) were synthesized by Genewiz (Suzhou, China) and cloned into a pUC57 vector, designated as pORFV/RP1. pORFV/RP1 plasmid DNA were extracted using Plasmid Mini kit I (Promega, USA) and then measured by Nanovue (GE lifescience). The DNA copy number was calculated using the following equation [18]: DNA copy number = (M× 6.02 × 10^23^ × 10^−9^)/(n × 660). The DNA standard was then aliquoted and stored at −80 °C until use.

### RPA primers and probe

ORFV-specific RPA primers and probes, based on the highly conserved DNA polymerase gene coding sequence of ORFV, were designed according to RPA guidelines from TwistDx (Cambridge, United Kingdom). All ORFV DNA polymerase gene coding sequence genes were retrieved from GenBank and multiple sequence alignment of the gene sequences were manually designed based on the ORFV DNA polymerase gene recommendation by TwistDx (Cambridge, UK). All primers and probes were synthesized by Sangon Biotech (Shanghai, China). Probes were synthesized with an inverse arrangement of fluorophore (6-carboxyfluorescein [FAM]), quencher (black hole quencher 1 [BHQ-1]), spacer(tetrahydrofuran spacer[THF]) and block elongation(phosphate[P]).

### Real-time qPCR assay

Real-time qPCR assay was performed with SYBR ® Select Master Mix on Aglient Technologies Stratagene Mx3005P thermocycler (Life technologies) as previously described [22]. The reactions were prepared as a 20 μL reaction volume containing SYBR® Select Master Mix (2X), the forward and reverse primers (10 μM, 1 μL each) and 2 μL of DNA template. The cycling parameters were as follows: 50 °C for 5 min, 95 °C for 10 min, 40 cycles at 95 °C for 15 s and 60 °C for 1 min. A melting curve analysis was performed using its specific melting temperature to verify the uniqueness of the amplified product. The data were analyzed using Mx3005P System software.

### Probe-based exo RPA assay

Exo RPA reactions were performed in a 50 μL volume using enzyme pellets from the TwistAmp exo kit (TwistDx, Cambridge, United Kingdom), which consisted of 27.5 μL rehydration buffer, 2 μL template DNA, 2.1 μL of forward and reverse primers (10 μM, BGI.tech), 0.6 μL of RPA exo probe (10 μM, Sangon Biotech), 11.2 μL of ddH2O and 2.5 μL of magnesium acetate (280 mM). The assay was performed on Aglient Technologies Stratagene Mx3005P thermocycler (Life technologies) for 60 cycles at 40 °C for 20s. The reaction was completed in 20 min. Optimal reaction conditions were defined after testing different incubation temperatures (39 to 42 °C), as well as different concentrations of template (0.5 μL to 2 μL) and magnesium acetate (1 μL to 2.5 μL). During initial experiments, the original 50 μL volume of the RPA reaction was successfully reduced to 25 μL. Fluorescence intensity of FAM was determined every 20 s. A sample was deemed positive if all replicates were three and a half standard deviations (3.5SD) above the background during a defined time range (i.e. after 19 to 20 min of amplification). A threshold time range of 0 to 4 min and 30 s was used.

### Statistic analysis

Data values are provided as the mean and standard deviation (SD). Statistical analysis was performed using PRISM 5.0 software (GraphPad Software, USA).

## Results

### Sensitivity and specificity of ORFV exo RPA assay

Initially in order to determine the most efficient primer pair for ORFV exo RPA assay, three forward primers (F1-F3) and reverse primers (R1-R3) based on different regions of ORFV DNA polymerase gene were designed (Table 1). Nine different combination of primers (i.e. F1/R1, F1/R2, F1/R3, F2/R1, F2/R2 F2/R3, F3/R1, F3/R2, F3/R3) were then tested with the exo probe ORFV RPA P on 10^4^ genome copies of standard DNA. The result showed that the primer set F1/R1 yielded the highest efficiency of amplification (data was shown in Additional file 1: Figure S1). Therefore, this pair of primers were employed in ORFV exo RPA assay for further validation.

**Table 1:** RPA primers and probes designed in this study

Name	Sequence (5’ –3’)	Genome location(U33419.1)
ORFVRPA F1	CTAGTAAGCTGTTCGAGATCACCTTGTTCATCATG	4465-4499
ORFV RPA F2	TTCCGACGGACGTATGAATATGTCCATGGTGAACG	4530-4564
ORFV RPA F3	CGTATGAATATGTCCATGGTGAACGATGTACCAAC	4540-4574
ORFV RPA R1	AGCGTTCATTCAATTCATGTCTGAGGTAAACGGCA	4702-4736
ORFV RPA R2	GACCACGTAAAAGTGGTGTTCGAAAAACTTCACAA	4651-4685
ORFV RPA R3	GGTAAACGGCAATGATGTTCGTGACAAAGACCACG	4679-4713
ORFV RPA P	AACGTATCCCATGCAGTAAAGCATAGTCCG	4582-4631
	(FAM-dT)C(THF)C(BHQ1-dT)TATAAACTCAGGAAC-p	

ORFV RPA F and R, RPA primer; ORFV RPA P, RPA Exo probe; BHQ1-dT, thymidine nucleotide carrying Black Hole Quencher 1; THF, tetrahydrofuran spacer; FAM-dT, thymidine nucleotide carrying fluorescein; P (phosphate), block elongation

**Fig. 1:**
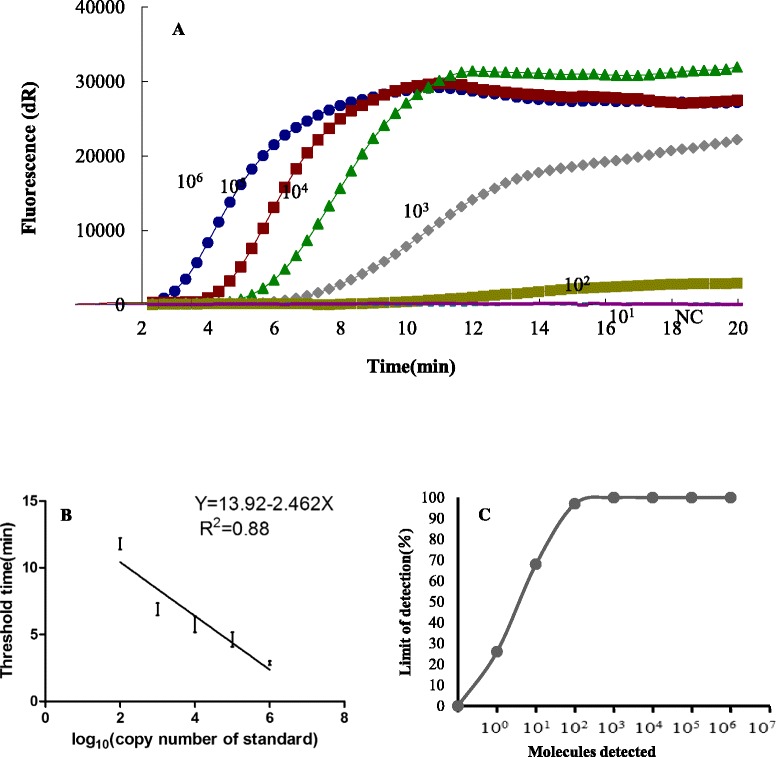
Performance of the ORFV exo RPA assay. **a** Amplification curve of ORFV exo RPA assay over time using a dilution range of 10^6^ to 10^1^ copies/reaction of ORFV. NC represent negative control. **b** Reproducibility of the ORFV exo RPA assay. The threshold time is represented as the mean ± standard deviation (SD). The standard regression line was generated based on 8 data sets (**c**) Probit regression analysis using Statistics software was done on data from the eight runs of ORFV exo RPA assay. The limit of detection at 95 % probability is depicted by a triangle

To test the dynamic range of ORFV exo RPA assay, standard DNA from pORFV/RP1 was diluted 10-fold, ranging from 10^1^ - 10^6^ genome copies per reaction and then tested by ORFV exo RPA assay. Every run was repeated 8 times. The threshold time was plotted against log (detected molecules), and a semi-log regression was calculated using PRISM 5.0 software (GraphPad Software, USA). As shown in Fig 1, the dynamic detection range of the assay spans 5 logs ranging from 6 to 2 log copies per reaction, with the corresponding threshold time ranging from 2 min at 10^6^ copies/reaction to 10 min at 10^2^ copies/reaction. This result indicates that ORFV exo RPA assay has a wide dynamic range for quantifying target DNA (Fig. 1a, b). The detection limit of ORFV exo RPA assay at 95 % probability was 10^2^ copies per reaction (probit analysis, p ≤ 0.05) (Fig 1c). The specificity of ORFV exo RPA assay was determined by cross detection of other viruses that infect sheep and goat epithelium or mucus including FMDV serotypes O, A and Asia 1, PPRV and Capripox virus. No cross detections were observed and all three different ORFV strains could be detected by the developed ORFV exo RPA assay (Table 2).

**Table 2:** Evaluation of the specificity of ORFV exo PRA assay

Virus family	Virus specie	Virus strain	exo RPA	qPCR
Poxviridae	ORFV	ORFV/Vaccine/CHA	5 min	12(CT)
	ORFV	ORFV/Gansu/CHA	3 min	9(CT)
	ORFV	ORFV/HB/CHA	4 min	11(CT)
Poxviridae other than ORFV	Capripox	Capripox virus/China Vaccine	neg	neg
	Capripox	Capripox virus/Henan/CHA	neg	neg
Paramyxovirinae	PPRV	Nigeria 75/1	neg	neg
Picornaviridae	FMDV	FMDV/O/CHA	neg	neg
	FMDV	FMDV/A/CHA	neg	neg
	FMDV	FMDV/Asia1/CHA	neg	neg

neg: negative

**Fig. 2:**
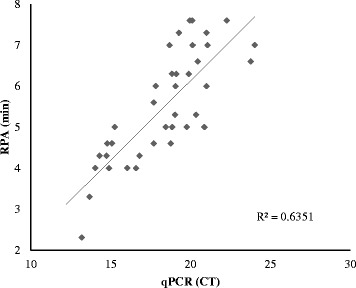
Comparison between performances of ORFV exo RPA assay and real-time ORFV qPCR assay on samples of ORFV-infected cells (*n* = 15) and spiked tissues lysates (*n* = 24). Linear regression analysis of the exo RPA threshold time (y axis) and qPCR cycle threshold (CT) values (x axis) were determined by Excel software

To further determine the sensitivity of ORFV exo RPA assay, extracted ORFV DNA from ORFV-infected A549 cells collected at 12, 24, 36, 48 and 60 hpi were evaluated (2 samples each time point). The amplification of ORFV DNA in ORFV-infected A549 cells was shown successfully from as early as 12 hpi, with threshold time (min) ranging from 6.8 ± 0.3 at 12hpi to 4.3 ± 0.4 at 60hpi. There was no amplification detected in the non-infected A549 cells used as negative control for this assay even through the threshold time (min) was greater than 30 min. These results were 100 % in agreement with these of real-time ORFV qPCR assay (a CT value ranging from 23.98 ± 0.2 to 14.8 ± 0.1). As a further investigation to verify sensitivity, the developed ORFV exo RPA assay was further tested using samples of ORFV-spiked tissues lysates (*n* = 24). Results showed that all the virus-spiked samples were positive for ORFV DNA, with threshold time (min) ranging from 7.1 ± 0.4 to 5.8 ± 1.1. There was no amplification detected in the non-virus-spiked samples despite threshold time being over 30 min. These results were 100 % confirmed by real-time ORFV qPCR assay (a CT value ranging from 18.4 ± 0.8 to 21 ± .0.9). Both assays showed the same performance on the samples above and correlation was found between values of the cycle threshold (qPCR) and threshold time (PRA) (R squared 0.64, Fig. 2).

### Performance of ORFV exo RPA assay on clinical samples

The practicality and efficiency of the ORFV exo RPA assay was evaluated with clinical samples and then compared with real-time ORFV qPCR assay. The clinical samples included twenty two samples (*n* = 22) collected from suspected cases of Orf, eight nasal swabs collected from experimentally infected sheep and five samples obtained from healthy goats. There was no amplification detected in samples (*n* = 5) obtained from healthy goats even through threshold time (min) exceeded 30 min. Nasal swabs (*n* = 8) were positive for ORFV through ORFV exo RPA assay (average threshold time = 6.3 ± 1.3, ranging from 4.6 to 8.6). Both results above were 100 % in agreement with these of the real-time ORFV qPCR (average CT value 21.3 ± 0.3, ranging from 16.6 to 26.2). Of 22 samples collected from suspected cases of the Orf, six samples were determined to be positive by ORFV exo RPA assay (threshold time ranging from 4.3 to 7.3) while eight samples were found to be positive by real-time ORFV qPCR assay (CT value ranging from 15.3 to 32.8). For the two samples found negative by ORFV exo RPA assay, the PCR assay CT value were the lowest (31.8 and 32.8, respectively). Based on a total of 35 samples examined, the sensitivity and the specificity of ORFV exo RPA assay for identification of ORFV was 86 % and 100 % respectively when compared to real-time ORFV qPCR (Table 3).

**Table 3:** Comparison of ORFV exo RPA assay with qPCR assay on clinical samples^a^

		qPCR		
		Positive	Negative	
RPA	Positive	14	0	14
	Negative	2	19	21
		16	19	35

^a^Samples include twenty two samples collected from suspected cases of the orf, eight nasal swabs collected from experimentally infected sheep and five samples obtained from healthy goats. All samples either ORFV or no viral DNAs detected

## Discussion

In the present study, a novel fluorescent probe-based RPA assay (ORFV exo RPA assay) was developed and evaluated for detection of ORFV. We demonstrated that the developed ORFV exo RPA assay could detect ORFV at a copy as low as 100 genome copies/reaction, furthermore the results have demonstrated that the developed ORFV RPA exo assay only specifically detects ORFV.

Our data from ORFV-infected A549 cell line has shown that ORFV DNA levels increases over time after inoculation, which indicates the potential capability for quantification of the virus in samples and also that A549 cell line could be a proper cell line for ORFV replication and propagation. No amplification was observed in non-ORFV infected cells/homogenizing tissue samples, this indicates the high specificity of ORFV exo RPA assay in this study. The apparent sensitivity of ORFV exo RPA assay for identification of ORFV in clinical samples was found to be 86 % when compared to real-time ORFV qPCR assay. We believe that this is due to the slightly lower sensitivity of ORFV exo RPA assay and the fact that those two samples were shown to be only weakly positive by real-time ORFV qPCR (a Ct value > 31). The Ct value of such ORFV qPCR positive/ORFV exo RPA negative samples were lower than that of the ORFV qPCR-positive/ORFV exo RPA positive samples. The results taken together indicate that the sensitivity and specificity of ORFV exo RPA assay, using F1/R1 primer set based on the region of ORFV DNA polymerase gene sequences, is comparable to real-time ORFV qPCR assay for detection of ORFV. A recent study showed that the sensitivity of RPA remains slightly lower than that of LAMP assay [23]. In comparison to RT-PCR and LAMP assays, RPA is a relatively novel and less evaluated technology. It is participated that its sensitivity could be further improved as the technology develops and the reaction conditions including RPA primers are fully optimized and evaluated. It is worth mentioning that maximal running time the developed ORFV exo RPA assay required is 30 min, regardless of the viral concentration present in samples as long as it is above the detection limit (i.e. > 100 copies per reaction).

In comparison, to the loop-mediated isothermal amplification (LAMP) technology, which requires a larger set of six primers, a higher temperature (62 °C) and a longer run time, the developed ORFV exo RPA assay is simpler and more rapid. The result can be obtained in less than 20 min and a lower reaction temperature of 37 ~ 39 °C is needed, which is an advantage with regard to miniaturization and integration into pen-side tests. In addition, it has high specificity due to the fact that RPA uses specific detection probes, like real-time PCR assay does, while LAMP uses nonspecific intercalating fluorophores [18, 24, 25]. Unlike LAMP assay,the specificity of the real-time fluorescent probe-based RPA assay based on TwistAmp exo kit, which was used in this study, cannot be checked in agarose gel, because the exonuclease present in the reaction mixture digests most of the amplified product once amplification has ceased. However, amplicons can be checked in agarose gel when TwistAmp basic kit or TwistAmp nfo kit in which the exonuclease is not included, is used, as we demonstrated with a specific band of amplified ORFV product in the gel electrophoresis based on TwistAmp basic kit (Additional file 2: Figure S2). Therefore, RPA has good flexibility in adaption into various detection systems.

## Conclusions

The ORFV exo RPA assay described is sensitive and specific for rapid detection of ORFV within less than 30 min. The results are encouraging but the assay must be validated by analysis of a larger number of samples from animals infected with ORFV and with different strains before such an assay can be considered for routine diagnostic use.

## Additional files

Additional file 1:
**Figure S1.** Three forward primes (F1-F3), one probe and three reverse primers (R1-R3) were tested to select combinations yielding the highest analytical RPA sensitivity. The amplification results of nine different combination of primers with the exo probe ORFV RPA P (i.e. 1: F1/R1, 2: F2/R2, 3: F1/R2, 4: F2/R1, 5: F1/R3, 6: F2/R3, 7: F3/R1,8: F3/R2,9: F3/R3, 10: NC) were shown in the figure. Standard DNA of 10^4^ genome copies were used as template. (DOCX 24 kb) Additional file 2:
**Figure S2.** Analysis of the sensitivity of the recombinase polymerase amplification in agarose gel. TwistAmp basic Kit was used in this reaction. A serial dilution of the ORFV DNA standard plasmids. Positive RPA reaction products (273 bp) can be detected on a stained agarose gel (2 %) (A). NC represent negative control. (DOCX 38 kb)

## Abbreviations

FBS: fetal bovine serum
FMDV: foot-and-mouth disease virus
LAMP: loop-mediated isothermal amplification
MEM: minimal essential medium
ORFV: orf virus
PBS: phosphate-buffered saline
PPRV: peste des petits ruminants virus
qPCR: quantitative PCR
RPA: recombinase polymerase amplification
