# Supplementary figures and images for: Development of a fluorescent probe-based recombinase polymerase amplification assay for rapid detection of Orf virus
Source: Virol J. 2015 Dec 2;12:206. doi: 10.1186/s12985-015-0440-z (PMC4668657; doi:10.1186/s12985-015-0440-z)

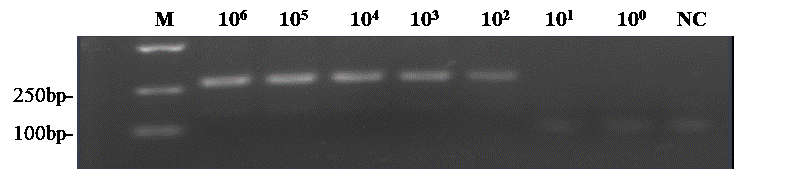


Figure S2

Supplement: Additional file 2: — Figure S2. Analysis of the sensitivity of the recombinase polymerase amplification in agarose gel. TwistAmp basic Kit was used in this reaction. A serial dilution of the ORFV DNA standard plasmids. Positive RPA reaction products (273 bp) can be detected on a stained agarose gel (2 %) (A). NC represent negative control. (DOCX 38 kb) [file 12985_2015_440_MOESM2_ESM.docx]
